# Locating and Imaging through Scattering Medium in a Large Depth

**DOI:** 10.3390/s21010090

**Published:** 2020-12-25

**Authors:** Shuo Zhu, Enlai Guo, Qianying Cui, Lianfa Bai, Jing Han, Dongliang Zheng

**Affiliations:** Jiangsu Key Laboratory of Spectral Imaging and Intelligent Sense, Nanjing University of Science and Technology, Nanjing 210094, China; zhushuo@njust.edu.cn (S.Z.); njustgel@163.com (E.G.); CuiQianying@njust.edu.cn (Q.C.); blf@njust.edu.cn (L.B.); dlzheng@njust.edu.cn (D.Z.)

**Keywords:** inverse scattering, locating and imaging through scattering medium, deep learning

## Abstract

Scattering medium brings great difficulties to locate and reconstruct objects especially when the objects are distributed in different positions. In this paper, a novel physics and learning-heuristic method is presented to locate and image the object through a strong scattering medium. A novel physics-informed framework, named DINet, is constructed to predict the depth and the image of the hidden object from the captured speckle pattern. With the phase-space constraint and the efficient network structure, the proposed method enables to locate the object with a depth mean error less than 0.05 mm, and image the object with an average peak signal-to-noise ratio (PSNR) above 24 dB, ranging from 350 mm to 1150 mm. The constructed DINet firstly solves the problem of quantitative locating and imaging via a single speckle pattern in a large depth. Comparing with the traditional methods, it paves the way to the practical applications requiring multi-physics through scattering media.

## 1. Introduction

Reconstructing the image and locating the depth of the object through the scattering medium play an important role in atmospheric and biomedical optics [1,2]. Sensing the objects in scattering is a classical problem with a complex mapping principle. The scattering media disrupts original information of the objects, it brings difficulty to imaging and locates objects through scattering medium in a large depth [3]. Nowadays, the object recovery mainly focuses on imaging objects in the camera sensor and locating the depth of hidden objects. Some methods have been put forward to solve the scattering imaging problem, such as single-pixel imaging [4,5], time-gated holographic imaging [6,7], wavefront shaping imaging [8,9,10] and speckle correlation imaging [11,12,13,14,15]. However, the use of compressive sensing with single-pixel imaging is limited to scenes that are sparse on the chosen basis [4], the time-gated holographic imaging is mainly focusing on detecting ballistic and near-forward scattered photos [7], the state-of-the-art devices cannot shape the complex-valued wavefront precisely via wavefront shaping methods [16] and the speckle correlation methods have a strict limitation on the field of view [11,12]. Instead of building a complex physical model, deep learning (DL) is efficient to solve complex mapping relationships, which can generate an optimized model driven by a large-scale dataset [17]. For imaging through scattering media, the DL methods have been successfully demonstrated to reconstruct objects through ground glasses, multimode fibers and fat emulsion with high quality and efficiency [16,18,19,20,21,22].

The image restoration of a hidden object is the main focus of the above research works, rather than other physical information, for example, the location and the size of the hidden object. The observation and ranging of the celestial bodies can help the astronomers make a good sense of the universe. The precise locating and imaging for biological tissues is the key information to analyses and research for biologists. Thus, the object ranging and locating are essential to the atmosphere or biological applications. To date, some techniques can acquire the hidden object depth via WFT-based (Windowed Fourier Transforms) memory effect measurement in phase space [23], PSF manipulation [24], chromatic aberration compensation [25] and coherence gating [26]. In order to obtain the depth information, the traditional physical methods are realized by indirectly mapping the relationship between the speckle patterns and the depth of the objects. The chromatic aberration compensation and coherence gating methods have good performance among traditional methods, which need additional optical reference arm to provide depth-related reference information. The reference arm configuration makes the experimental arrangement and adjustment more complicated. Besides, these depth detection methods are difficult to build complete physical models for obtaining absolute depth. These critical requirements to acquire the physical information in these detection methods restrict their wide application in practice. In which, the reported maximum range for locating and imaging is 500.07 mm via coherence gating [26]. Further expansion of the depth detection capabilities is meaningful for expanding practical applications. However, the DL method on depth detection is limited with physical instruction and effective network structure. The depth information of hidden objects cannot be measured efficiently, with or without prior information, due to the scattering disruption and the model ability. Thus, selecting an efficient neural network structure and designing the DL framework with physical prior are the way to solve the inverse problem in scattering.

In this paper, for the first time, a novel framework is proposed to realize depth prediction and image reconstruction (DINet) from a single speckle pattern, which is a dual-path network providing different attributes. Unlike usual DL applications, the information distribution or degradation degree is more serious in the case of scattering. It is hard to extract effective features from speckle patterns directly via usual DL methods. Thus, DINet needs the great capability of physical mapping and data mining to obtain useful information from the scattered light. DINet with a phase-space informed locating-path network and an effective Encoder-Decoder structure imaging-path network is proposed to solve the challenge of depth prediction and image reconstruction in a large depth. DINet can also simplify the hardware requirements and experimental process by removing the reference arm configuration. To the best of our knowledge, this is the first model that solves the problem of quantitative locating and imaging in a field up to 1150 mm through a strong scattering medium, and the maximum depth mean error is about 0.1 mm nearby the 1150 mm depth.

## 2. Principle

**Figure 1:**
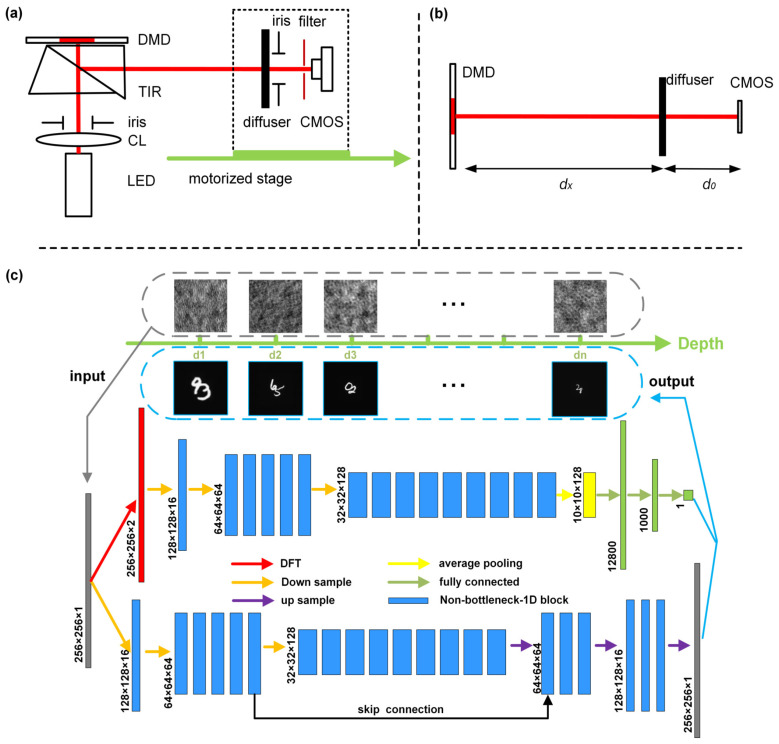
Systematic configuration. (**a**) Experimental arrangement. CL, collimating lens; TIR, total internal reflection prism; DMD, digital micro-mirror device. (**b**) Description of imaging distance with optical path unfold. (**c**) Schematic diagram of DINet Architecture.

The end-to-end DINet is proposed to learn a statistical model relating to the speckle patterns generated in different depths. The practical systematic configuration, which is designed to collect the experimental data including the speckle patterns and the distance between object and diffuser, is drawn schematically in Figure 1a. As for Figure 1b, it is the description of locating distance with the optical path unfolded. The content structure of DINet is shown in Figure 1c. A single speckle pattern is the input of the dual-path network, which produces a depth value by the locating-path network and a recovered 256×256 image with the imaging-path network.

### 2.1. Basics of Locating and Imaging

Phase-space measurement of scattered light can provide the features for depth information calibration [27]. Phase-space optics contains the spatial and spatial frequency information, which allows the visualization of space depth information [23]. The Wigner distribution function (WDF) can be used to describe the phase space features as

(1) $$f\left(r,k\right)=\int \langle \psi^{*}\left(r+\frac{\xi }{2}\right)\psi \left(r-\frac{\xi }{2}\right)\rangle e^{ik\xi }d\xi ,$$

where r=(x,y) and $k=(k_{x},k_{y})$ are the two-dimensional spatial and spatial frequency vectors, ξ is the transverse spatial variable centered on *r*, and ψ(r) is the wave spread function [28,29]. Due to the visible phenomenon after post-processing, the depth variation makes the phase-space features change regularly, and the fitted slope of speckle patterns, *A*, is related to variable depth linearly as $A\propto 1/d_{x}$ [23]. Thus, the DFT process of speckle patterns can help the locating-path network to extract the distance features for depth prediction. DINet can regress the depth value via effective data mining and powerful fitting capability, which can optimize the complex mapping relationships as:

(2) $$d_{x}=ϝ\left(S,d_{0},\lambda \right),$$

where ϝ is the mapping function, *S* is the speckle pattern, $d_{0}$ is the distance between the CMOS and the scattering medium, and λ is the wavelength of the light source.

To restore the object through the scattering medium is a highly ill-posed and prone noise-induced problem. The prior knowledge can be introduced to optimize the inverse problem, for example, the physics-prior features and the ground truth. The optimization function can be expressed as

(3) $$\hat{O}={\mathrm{argmin}}_{O}∥{G(O)-M∥}^{2}+\alpha \lambda R(O),$$

where *G* is the forward operator in the scattering system, *O* is the object through the scattering medium, *M* is the raw intensity image, R(O) is the regularization term, and $\hat{O}$ is the estimate of the object. The DL method is good at optimizing inverse problems using a large amount of data and mining the physical mapping relationship, which can reconstruct the object with high imaging quality in case of scattering [18,22].

### 2.2. DL Framework Implementation

The key structure of the multi-task network is based on Efficient Residual Factorized (ERF) layers [30], which is a residual architecture with reducing the computational costs and remaining the remarkable accuracy. The locating-path consists of Discrete Fourier Transform (DFT), encoder part and fully connected layers to extract the location features and regress the depth values. The necessity of the DFT step is presented in the Appendix A. The imaging-path follows the encoder-decoder architecture with modification of the long-range skip connection to improve the reconstruction quality [22]. The details of DINet are provided in Appendix B.

To train DINet, the mean absolute error (MAE) and the mean squared error (MSE) are used as the loss function. The MAE for the locating-path and the MSE for the imaging-path have a good performance in this multi-task. The loss functions are calculated as

(4) $$Loss_{D}=\frac{1}{N}\sum_{i}^{N}\left|D_{i}-D_{gt}\right|,$$

(5) $$Loss_{I}=\frac{1}{N}\sum_{i}^{N}{\left|I_{i}-I_{gt}\right|}^{2},$$

where $D_{i}$ and $D_{gt}$ are the predicted values and true values, $I_{i}$ and $I_{gt}$ are the reconstructed images and ground truths, respectively; *i* is the index number in the training dataset, and *N* is the mini-batch size. Two sub-networks can be trained synchronously via $Loss_{DI}$, which defined as

(6) $$Loss_{DI}=Loss_{D}+Loss_{I}.$$

The locating-path network and the imaging-path network are sharing the same input instead of other layers in the DINet framework. Thus, the locating-path network and the imaging-path network are two independent networks. If only one particular function is needed, the sub-path network can also be trained and work independently.

### 2.3. Setup and Data Acquisition

**Figure 2:**
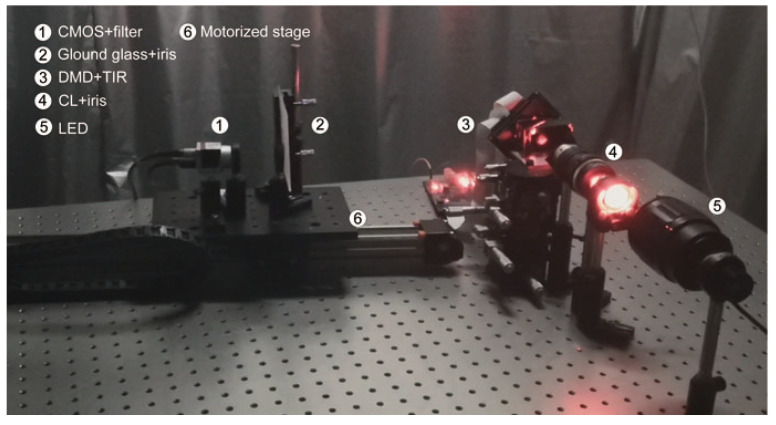
Schematic of Speckle collecting setups.

The proposed DINet is tested on real optical datasets and the datasets acquisition system is displayed in the Figure 2. A digital micro-mirror device (DMD) (pixel count: 1024 × 768, pixel pitch: 13.68 µm) is employed to display handwritten digits, which are selected as object images from the MINIST database on its surface. A TIR prism is employed to fold light path for capturing the patterns conveniently. A ground glass (Thorlabs, Newton, NJ, USA, DG100X100-220-N-BK7, 220 Grit) is selected as the diffuser with strong scattering so that objects are completely hidden. The LED (Thorlabs, M625L4) combined with a filter (Thorlabs, FL632.8-1, central wavelength: 632.8 ± 0.2 nm) is designed as the narrow band partially coherent light source for the experimental arrangement. The speckle patterns corresponding to different positions can be obtained by moving the motorized stage. As shown in Figure 1b, the optical path can be unfolded from TIR prism between the DMD and the CMOS (Balser, acA1920-155 um). The value of $d_{0}$ is 80 mm which is the distance between the ground glass and the CMOS camera working surface. The devices in the dashed box are fixed relatively to ensure a constent $d_{0}$. Thus, the experimental setup within the dashed box are moved within the working stroke by the motorized stage to get the variable depth of the hidden objects, and the effective working distance between the diffuser and the CMOS is ranging from 350 mm to 1150 mm. If necessary, DINet can be also used in the different positions, even more than 1150 mm or other specific scenarios.

## 3. Results and Analysis

For the training of DINet, 1100 speckle patterns are recorded at each depth and selecting 1000 speckle patterns as the training data, 50 speckle patterns, and another 50 speckle patterns are selected as validation data and testing data, respectively. The training set is processed with a mini-batch size of 32. Each model is trained with 400 epochs by Adam optimizer for up to 8 h. The learning rate starts from $5\times 10^{-4}$ in the first 200 epochs to $5\times 10^{-5}$ for the final 200 epochs. The training and testing environment is PyTorch 1.3.1 and CUDA 10.1 version under ubuntu 16.04. The hardware specifications for the workstation is composed of a single NVIDIA GeForce Titan RTX graphics unit and the intel Core i9-9940X central processing unit.

### 3.1. Quantitative Evaluation in the Whole Working Stroke

**Figure 3:**
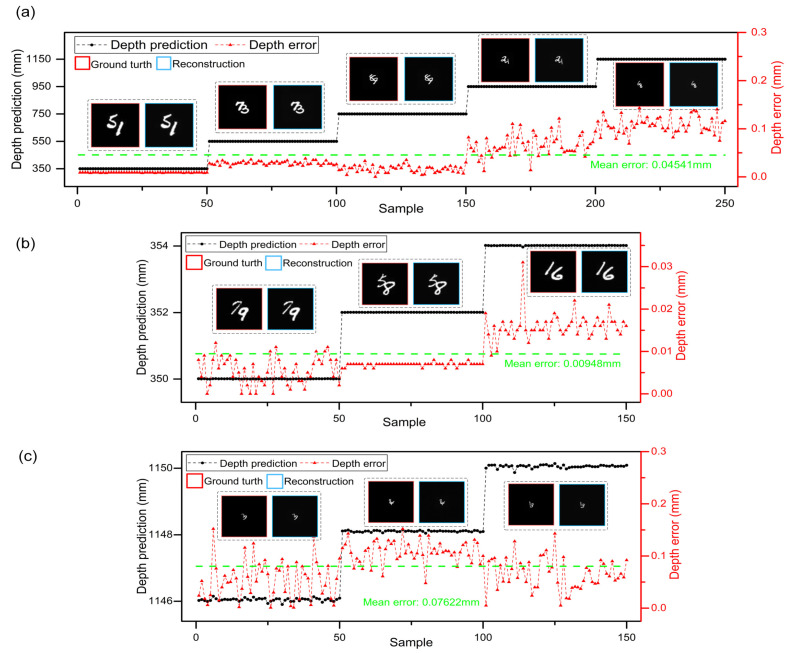
Quantitative evaluation for the DINet. (**a**) The depth predictions and errors in 5 positions. An object ground truth and corresponding reconstruction are shown in each sample range. (**b**,**c**) are the resolution capability of the DINet in different ranges.

To quantitatively evaluate the locating and imaging performance of DINet, the MAE and peak signal-to-noise ratio (PSNR) are employed to measure the locating accuracy and imaging quality. The multi-task results of untrained samples in the whole working stroke are shown in Figure 3a, which include the locating testing results and imaging examples with corresponding ground truth. The distance of the object relatively moves from 350 mm to 1150 mm with a stride of 200 mm. The abscissa is the testing sample sequence and each depth has 50 untrained samples. Obviously, DINet can regress the depth of different distribution and restore the hidden object. The locating accuracy and error distribution are represented clearly via comparing with the mean depth error, which is 0.04541 mm indicated with the green dash line. From the depth error distribution, the locating accuracy is reducing with distance increasing. Meanwhile, the result of the imaging-path evaluated via MAE and PSNR is drawn in the Table 1 and the average PSNR of imaging result is up to 24.71 dB in the whole working stroke. As the distance increases, the size of imaging results is decreasing proportionally and the imaging quality is also reducing slightly.

DINet has subdivision capability to locate and reconstruct from large stride to slight range. Both ends of the working stroke are selected to test DINet with changing the stride to 2 mm. As shown in Figure 3b,c, DINet can obtain higher locating accuracy and better imaging quality in the beginning stroke (350–354 mm) on the multi-task. Corresponding to the beginning stroke, DINet can also complete the multi-task during the ending stroke (1146–1150 mm) of the subdivided working stroke. Furthermore, the refining capacity of the ending stroke is lower than the beginning in case of the same stride, with a mean error declining from 0.00948 mm to 0.07622 mm. The results agree with the above analysis of the error distribution. To conclude, DINet has good performance in locating and imaging as shown in Figure 3 and the Table 2. However, with the distance increasing, the quantity of the collected speckle patterns is limited by CMOS sensitivity. Besides, more system noise will be introduced, such as stray light and stage collimation error. All of the above factors will result in a negative impact on locating accuracy and imaging quality. Furthermore, we also provide the additional imaging results which comparing with conventional Unet [31] and Dense-Unet [19], and the imaging-path of DINet has better imaging results through scattering medium in a large depth.

### 3.2. Depth-Resolved Results with Randomly Distributed Objects in a Plane and Different Sizes of the Objects

**Figure 4:**
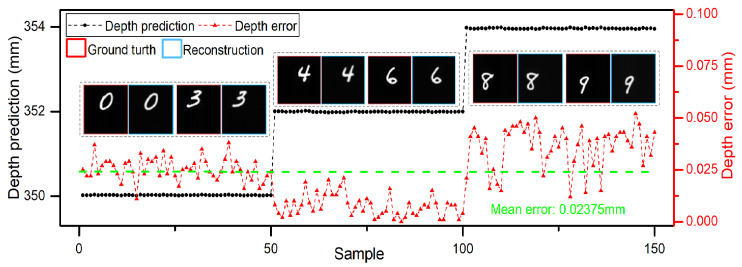
Locating and imaging results with no-fixed position objects in a plane.

When the objects are in the center of the planar, the DINet can locate and reconstruct objects accurately. Furthermore, DINet can also locate and reconstruct the objects with no-fixed position in a plane. As shown in Figure 4, single characters with no-fixed position were selected as hidden objects, DINet has the accurate locating capability with a depth mean error 0.02375 mm and can reconstruct the correct objects correspondingly. However, comparing to the objects with a fixed position in the center of the plane, the locating accuracy and imaging quality are decreasing slightly with randomly distributed objects. The experimental results with no-fixed position objects in a plane prove that the locating capability of DINet is relevant to the depth of the objects and DINet can also reconstruct the correct spatial position of the objects.

**Figure 5:**
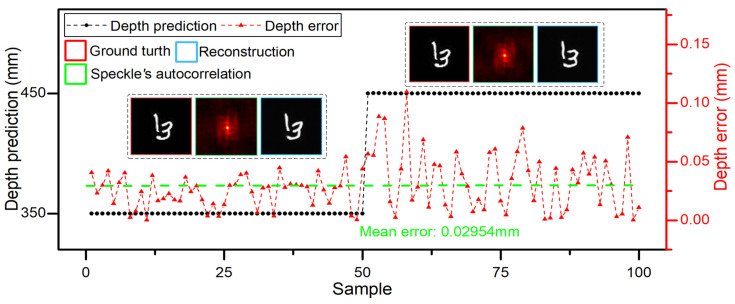
Locating and imaging results with the different object size and the same imaging size.

It should be further clarified that the size of ground truth captured without scattering medium is proportional to the distance between the CMOS and the DMD in the experimental results corresponding to Figure 3a. Meanwhile, the size of speckle autocorrelation through the scattering medium is also proportional to the distance between the diffuser and the DMD [32]. However, the distance dx is the only variable related to the recording depth of the speckle patterns via DINet. In order to obtain a similar imaging size, the size of objects on the DMD is adjusted according to the system magnification with different $d_{x}$. Thus, the 80×80 pixel objects and the 102×102 pixel objects are selected in the 350 mm depth and the 450 mm depth, respectively. The depth can be measured accurately even if the object size is scaled on the DMD, the regressed depth and reconstructed image are shown in Figure 5. The experimental result with multiple object sizes proves that the depth-resolved capability of DINet is mainly relevant to the depth of the object and has nothing with the size of the object. Both the depth of the object and structure information is contained in a single speckle image, which can be separated via the DL method.

### 3.3. Generalization Capability

When the object movement is within a certain range of depth, the speckle pattern of a diffuser is relatively deterministic along the z-direction, and this character is the basis of the PSF manipulation method. Once the movement range is a too large, the correlation of speckle will be greatly reduced which results in the maximum range is 36.6 mm by the PSF manipulation method [24]. We utilize the DINet to extend the efficient stroke up to 1150 mm with the depth mean error 0.05 mm. Furthermore, in order to use the conclusion along the z-direction from [24] which the adjacent speckle pattern has a certain relevance along the z-direction, we provide results of DINet about the generalization capability with different sampling intervals.The more relevant speckles along the z-direction with a 2 mm stride can provide the better generalization capability of locating unknown depth, and the too large stride (e.g., 200 mm) will cause the relatively poor generalization capability in unknown depth. On the other hand, different scenes have different acceptable margin of errors, the proper sampling interval can be selected as the training dataset.

**Figure 6:**
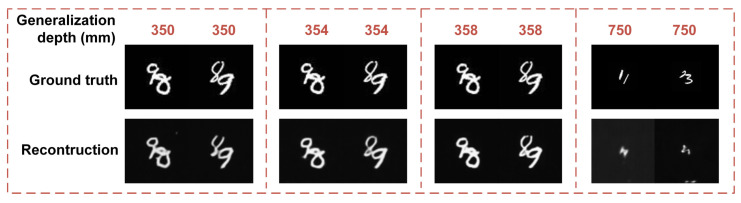
Reconstruction results in generalization depth.

As shown in Table 2, DINet has a different performance with the distribution of depth and step size. The accuracy of the generalized depth in 354 mm is better than 350 mm and 358 mm with four training depths. And the locating generalization performance of 2 mm stride is much better than 200 mm. From Figure 6, the imaging-path network can also restore the hidden objects in generalized depth. The quality of reconstruction in 354 mm is better than 350 mm and 358 mm, and the imaging results of 2 mm stride are much better than 200 mm. The generalization of missing distance in the middle is better than the marginal and the smaller sampling step is better than the large relatively. The results of depth generalization capability can also be explained from the aspect of data acquisition, which corresponds to the distribution of uniform sampling and the effective interval of sampling points. Thus, to refine the stride and expand the depth of coverage can improve the generalization performance of the locating accuracy and imaging quality. DINet can obtain relatively accurate generalization via the subdivision ability of dense measurement and extending the coverage of distance. The multi-modal measurement technology through a single speckle pattern based on DINet has potential in practical applications, for example, removing the effect of inclement weather conditions to produce a photo and a depth map in fog. The capability of depth generalization makes DINet pave the way to practical applications.

## 4. Discussion

According to the experimental results, several discussions are presented as followed:

(i) DINet is a novel framework that has good performance in locating and imaging through scattering medium in a large depth. This method can obtain accurate depth and imaging results in a large depth ranging from 350 mm to 1150 mm in strong scattering, with a mean error less than 0.05 mm in depth prediction and an average PSNR above 24 dB in image reconstruction.
(ii) DINet has great robustness in depth-resolved capability and good generalization performance in unseen planar position and unseen axial depth. DINet can locate and reconstruct the correct objects with high accuracy, even for the no-fixed position objects in the plane in different depths. For the locating capability of DINet, it should be noted that the depth-resolved capability of DINet is mainly relevant to the depth of the object and has nothing with the size of the object. Meanwhile, DINet has depth generalization capability to regress the depth value and reconstruct the objects with an unseen depth in the training process.
(iii) It should be clarified that DINet has different performance in locating and imaging with different scenarios. The locating accuracy and imaging quality of DINet are limited with the sensitivity of the CMOS with different working distances and the complexity of the dataset. The depth generalization capability also depends on the sampling strategy and the distribution of the training dataset.

## 5. Conclusions

In this paper, a novel DL framework is designed for depth prediction and image reconstruction using a single speckle pattern. This method, which is not limited to the discussed two tasks, does not need a complex experimental setup and can be applied to measure other physical information, such as locating in-plane coordinates and hidden object classification. The proposed DINet technique opens up the way to multiple physical information measurement in practical application in case of scattering. However, DINet can only regress the plane depth value in the case of scattering. In the future, for pixel locating or three-dimensional measurement in scattering, the physics-prior knowledge can also be introduced to help us design the DL method.

## Figures and Tables

**Table 1:** Quantitative evaluation of reconstruction each position.

Depth Value (mm)		350	550	750	950	1150
Imaging-path of DINet [22]	MAE	0.0145	0.0155	0.0159	0.0168	0.0188
	PSNR (dB)	25.51	25.02	24.82	24.46	23.73
Unet [31]	MAE	0.0214	0.0203	0.0195	0.0196	0.0201
	PSNR (dB)	21.88	22.58	22.92	22.88	22.83
Dense-Unet [19]	MAE	0.0240	0.0243	0.0263	0.0274	0.0294
	PSNR (dB)	21.53	21.59	21.10	20.98	20.40

**Table 2:** Results of depth generalization.

Training Depth (mm)	Generalization Depth (mm)	Mean Error (mm)
352, 354, 356, 358	350	0.66128
350, 352, 356, 358	354	0.21434
350, 352, 354, 356	358	0.52759
350, 550, 950, 1150	750	48.3811

**Figure A1:**
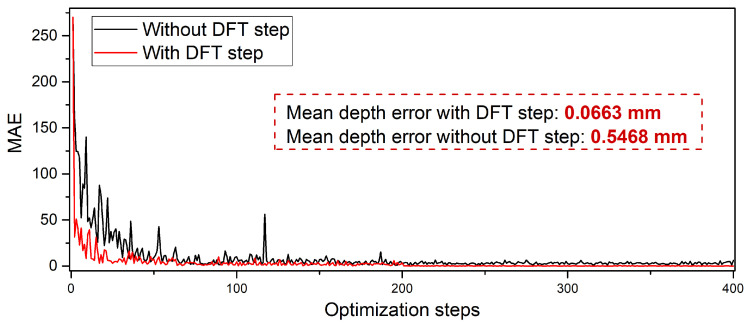
The influence of DFT step for locating accuracy and the speed of model convergence.

## Appendix A

The DFT step informed with phase-space-prior is crucial for locating through scattering media. Firstly, according to References [23,27], the phase-space measurement based on the windowed Fourier transform (WFT) can fit out the source distance from the diffuser as $A\propto 1/d_{x}$, *A* is the fitted slope of speckle patterns. Thus, the discrete Fourier transform (DFT) process of speckle patterns can help the locating-path network to extract the distance features for depth prediction. Secondly, the locating result with phase-space constraint with high accuracy in a large depth ranging from 350 mm to 1150 mm can also prove that the DFT features are useful for the locating-path network. As shown in Figure A1, we select the whole working stroke dataset to train and test. The neural network is competent to learn a DFT process, if we removed the DFT step, the speed of convergence is lower than before. Furthermore, comparing with the locating-path network with phase-space constraint, if we use the same initialization method and hyper-parameter with the same training strategy, the accuracy of the locating-path network without the DFT step is worse.

## Appendix B

In Table A1 and Table A2, the details of locating-path and imaging-path structure are provided, respectively. The table includes the input, output shapes, and the details of each CNN layer, where dilated denotes the size of dilated convolution, output size denotes the size of layer output, and Non-bt-1D denotes Non-bottleneck-1D block [30].

**Table A1:** Details of locating-path network.

		Type	Output Size
	Step 0	DFT Step	2×256×256
Encoder	Step 1	Downsample Layer	16×128×128
	Step 2	Downsample Layer	64×64×64
		5 × Non-bt-1D	64×64×64
	Step 3	Downsample Layer	128×32×32
		Non-bt-1D (dilated 2)	128×32×32
		Non-bt-1D (dilated 4)	128×32×32
		Non-bt-1D (dilated 6)	128×32×32
		Non-bt-1D (dilated 8)	128×32×32
		Non-bt-1D (dilated 2)	128×32×32
		Non-bt-1D (dilated 4)	128×32×32
		Non-bt-1D (dilated 6)	128×32×32
		Non-bt-1D (dilated 8)	128×32×32
Decoder	Step 4	Average pooling Layer	128×10×10
	Step 5	Fully connected Layer	12,800
		Fully connected Layer	1000
		Fully connected Layer	1

**Table A2:** Details of imaging-path network.

		Type	Output Size
Encoder	Step 0	Downsample Layer	16×128×128
	Step 1	Downsample Layer	64×64×64
		5 × Non-bt-1D	64×64×64
	Step 2	Downsample Layer	128×32×32
		Non-bt-1D (dilated 2)	128×32×32
		Non-bt-1D (dilated 4)	128×32×32
		Non-bt-1D (dilated 6)	128×32×32
		Non-bt-1D (dilated 8)	128×32×32
		Non-bt-1D (dilated 2)	128×32×32
		Non-bt-1D (dilated 4)	128×32×32
		Non-bt-1D (dilated 6)	128×32×32
		Non-bt-1D (dilated 8)	128×32×32
Decoder	Step 3	Upsample Layer	64×64×64
		2 × Non-bt-1D	64×64×64
	Step 4	Upsample Layer	16×128×128
		2 × Non-bt-1D	16×128×128
	Step 5	Upsample Layer	1×256×256
